# Women’s Ideas about the Health Effects of Household Air Pollution, Developed through Focus Group Discussions and Artwork in Southern Nepal

**DOI:** 10.3390/ijerph15020248

**Published:** 2018-02-01

**Authors:** Delan Devakumar, Zeshan Qureshi, Jenevieve Mannell, Manju Baruwal, Neha Sharma, Eva Rehfuess, Naomi M. Saville, Dharma S. Manandhar, David Osrin

**Affiliations:** 1 Institute for Global Health, University College London, 30 Guilford St, London WC1N 1EH, UK; zeshan.u.qureshi@gmail.com (Z.Q.); j.mannell@ucl.ac.uk (J.M.); n.saville@ucl.ac.uk (N.M.S.); d.osrin@ucl.ac.uk (D.O.); 2 Mother and Infant Research Activities, Kathmandu 44600, Nepal; manju.baruwal@gmail.com (M.B.); neha.sharma@mira.org.np (N.S.); dsm@mira.org.np (D.S.M.); 3 Institute of Medical Information Processing, Biometry and Epidemiology, Pettenkofer School of Public Health, LMU Munich, 81377 Munich, Germany; rehfuess@ibe.med.uni-muenchen.de

**Keywords:** household air pollution, focus groups, biomass, Nepal

## Abstract

Household air pollution is a major cause of ill health, but few solutions have been effective to date. While many quantitative studies have been conducted, few have explored the lived experiences and perceptions of women who do the cooking, and as a result are those most exposed to household air pollution. In this study, we worked with groups of home cooks, and sought to use art as a means of engaging them in discussions of how household air pollution from cooking affects their lives. In the Terai district of southern Nepal, we held four focus groups that included 26 local women from urban and peri-urban areas, as well as six local artists. The women then met approximately weekly over four months, and produced images related to air pollution. Transcripts from the focus groups were reviewed independently by two authors, who initially categorised data deductively to pre-defined nodes, and subsequently inductively reviewed emergent themes. Women identified a number of health effects from air pollution. The main physical effects related to the eye and the respiratory system, and women and young children were seen as most vulnerable. The psychosocial effects of air pollution included reduced food intake by women and lethargy. Suggested solutions included modifications to the cooking process, changing the location of stoves, and increasing ventilation. The main barriers were financial. The lived experiences of women in southern Nepal around the problem of air pollution offers a more nuanced and context-specific understanding of the perceptions and challenges of addressing air pollution, which can be used to inform future interventions.

## 1. Background

Biomass fuels are renewable organic materials such as animal dung, charcoal, coal, crops, and wood [1]. They are generally cheap and accessible, and—together with coal—are used by 2.7 billion people (41% of the world’s population), with little change over the last two decades [1,2,3]. In the most affected low and middle-income countries (LMICs), a majority of the population continues to cook with these fuels, including in Nepal, where it is estimated that 75% of households depend on them [4]. Biomass fuels burnt on inefficient stoves produce a complex mix of pollutants, including concentrations of particulate matter that are hundreds of times higher than those commonly encountered outdoors [5]. This so-called household air pollution is estimated to contribute to 2.9 million premature annual deaths worldwide, and 85.6 million disability-adjusted life years (DALYs) [6], and is experienced disproportionately by women, who do most of the cooking [7,8]. Air pollution is second highest cause of DALYs lost in Nepal [9]. Meta-analyses suggest that the use of solid fuel—of which biomass is an example—increases the risk of illnesses such as chronic obstructive pulmonary disease [10], pneumonia in children under five [11], lung cancer [12], and tuberculosis [13].

We have previously shown evidence of high air pollution concentrations in southern Nepal and high levels of exposure amongst children [14], and other groups have shown similarly high levels elsewhere in the country [15,16,17]. However, intervention through cleaner cooking stoves has been limited in scope [18,19,20]. The reasons for this are numerous. In addition to the technological characteristics and technical efficiency of alternative stoves, and financial, programmatic, and policy challenges, there are a number of additional barriers to uptake by potential users. These relate to the contexts in which they live and the realities of users’ everyday lives.

Local women represent a source of experiential knowledge, and—as primary cooks—are one of the primary target audiences for interventions to mitigate household air pollution. Although little has been done to involve them in the research process, they may be a key resource in identifying why the problem persists, and what potential solutions might be. A recent systematic review of factors influencing the uptake of cleaner stoves highlights the need for better understandings of the needs of socio-economically disadvantaged groups in particular [21]. However, even when potentially helpful messages are identified, the method of disseminating them is not always appropriate, and there may be a disconnect between communication and behaviour change [22]. For example, while the reduction of adverse health effects does appear to enable the adoption of cleaner cook stoves, perceptions that the smoke from current cooking methods has positive health benefits by controlling insects may also undermine the uptake of cleaner cooking methods in certain contexts [23]. This highlights the complexities of local understandings of the health benefits of improved stoves, and the need for further context-specific studies.

One approach to unravelling these complex issues is to use art as a means of generating discussion. Art has been used extensively as a tool in participatory global health research to engage communities and draw out implicit knowledge on health-related topics [24]. Drawings are frequently used as a means of engaging children in conversations about health-related issues [25,26], and the drawing of body maps and other visual techniques has been used with adults as a means of developing clear understandings of abstract concepts and shifting the balance of power from researcher to participants [27].

In our study in southern Nepal, we sought to discuss household air pollution with women who do the cooking, and are the primary people affected by it. Our aims were to explore their lived experiences and perceptions of the health effects of household air pollution through open-ended discussions. In order to draw out implicit understandings of how biomass fuels impact on health, we involved local artists in discussions to help illustrate the issues around household air pollution from cooking, and stimulate thinking about potential solutions.

## 2. Methods

### 2.1. Setting

We conducted a qualitative study in Janakpur, Dhanusha district, Nepal. Nepal is one of the world’s poorest countries, and Janakpur is a small city with little mechanised traffic or heavy industry, close to the border with India in the flat Terai region. The urban population lives mainly in concrete or mud and brick housing. The Nepal Census showed that 86% of households in Dhanusha district use either wood or animal dung as their usual source of fuel [4]. Previous research from Dhanusha district has shown that the stoves in households who use biomass tend to be simple, made from mud or clay, with no chimney or other adequate source of ventilation. The main biomass fuels are wood and animal dung formed into cakes with sticks or straw. Kitchens vary; they are usually in a separate room, but are often connected to the rest of the house [14].

The research was conducted within a larger study that investigated the effects of antenatal micronutrient supplementation and air pollution, with ethical approval granted by the Nepal Health Research Council (reference 51/2011) [14,28].

### 2.2. Sampling and Recruitment

A purposive sample of women living in Janakpur who were currently using biomass fuel for cooking were recruited from participants in the larger antenatal micronutrient supplement and air pollution study. For the larger study, women had been recruited from the obstetric unit of the local government hospital, and had been allocated randomly to receive multiple micronutrients or iron and folic acid during pregnancy [29]. Their children were then followed up to eight years of age [28]. Women from the city of Janakpur or nearby peri-urban areas were approached and asked to take part in this further study.

Local artists were recruited from the Janakpur Women’s Development Centre (JWDC). The JWDC is a co-operative organisation that produces local Maithili art. The female artists are mostly widows who come from poor communities nearby. As well as helping preserve their artistic heritage, the centre also works as a business that helps empower women.

All of the participants were from a similar background, and came from urban or peri-urban areas. They tended to be from middle socio-economic strata—not the poorest or the wealthiest—and all used biomass fuels. Fifty women were visited at home and invited to take part, of whom 26 attended the first focus ground discussions (FGDs), along with six artists. Each of the four groups had between five and eight people, as planned.

### 2.3. Data Collection

Focus group discussions (FGDs) were designed to serve two purposes: to gain insight into participants’ perceptions of air pollution from cooking through the use of art, and to guide a public engagement project. We conducted FGDs to obtain in-depth and nuanced insights about commonly shared perceptions of the health impacts of cooking. We held four initial FGDs in March and April 2013, conducted by two trained female Nepalese researchers, according to a topic guide. The guides covered cooking fuels and the reasons for choosing them, the health effects of air pollution, and possible ways to mitigate them. The facilitator allowed participants to talk about their experiences and opinions, and gain insights from each other with little input other than prompts for further information and the posing of questions when a topic was fully exhausted. One researcher facilitated the discussions and the other took notes. The artists were invited to participate in these initial discussions, since they were also women from the community who experienced similar issues. The research facilitator had previously conducted focus groups and women’s groups in the region, but had not met the participants beforehand. FGDs were held in a participant’s home or nearby. Participants provided written informed consent, and discussions were conducted in Nepali or the local language, Maithili.

After the first round of FGDs, the participants met with the JWDC artists in a series of workshops held approximately weekly over four months to illustrate their perceptions of the health effects of air pollution from cooking. The workshops were not formally recorded, but meeting notes were kept. Four different groups of women attempted to illustrate the problems they faced and possible solutions to them. All of the workshops were observed by one of the researchers, who took notes to guide the analysis. Prototype pictures related to how participants themselves saw the issues, and they themselves chose the final means of display. They decided to produce 11 large pictures with the intention of showing them around the city. A community meeting with local officials was held to identify locations to display the artworks, which were painted either directly on walls or on large billboards. They were placed in prominent locations around the city; for example, in the main public hospital, the railway station, and at a school.

### 2.4. Data Management and Analysis

Focus groups were audio recorded and transcribed and translated into English by two members of the research team. Two authors independently reviewed and coded the transcripts. One of whom also conducted the groups, was fluent in the local languages, and had a deep understanding of the problems faced by the local women. In contrast, the other was from the United Kingdom, and was not involved in conducting the discussions, which provided an alternative perspective to the analysis of the data.

A thematic analysis was used to analyze the data using both inductive and deductive approaches [30]. Initially, the transcripts were deductively separated into descriptive categories drawing from the initial topic guide, including perceived physical health effects (eye-related, respiratory, relating to pregnancy or children, and other), psychosocial health effects (cleanliness and financial implications), solutions (stoves, cooking process, and ventilation) and barriers (knowledge, resources, and helplessness). These descriptive categories were then further analysed inductively in order to develop bottom-up themes related to the lived experiences and women’s implicit understandings of perceived health effects, solutions, and barriers to realising these solutions. The FGDs were analysed as a single data source in order to draw out themes common across the different groups. While the images produced during the workshops were not analysed using semiotic or other visual analysis techniques, they did provide a means of ensuring group consensus and a shared understanding of the issues across the groups. The images produced by different groups were similar, with common themes despite different artists and different facilitators, suggesting that data saturation was achieved.

## 3. Results

We identified several common themes about the impacts of air pollution through our analysis of the FGDs on women’s lived experiences of cooking and health. These themes are presented below in order to describe how women interpreted and made sense of their experience of household air pollution, together with the illustrations produced by the artists during the workshops.

General conceptualisation of air pollution: ‘bad smells’.

In all four FGDs, air pollution was identified as an issue, and it was usually described as “bad smells”. Some felt that all ill health associated with household air pollution was related to the large amounts of dirt it created. Biomass cooking stoves were commonly mentioned as a source of household air pollution, although tobacco smoke was also mentioned. One participant explained the problem as “polluted air comes by cooking rice on mud stove that can affect … people living inside the house” (Focus Group 2 Participant 7 (FG2 P7). Smoke was thought to be a problem throughout the whole house, but particularly in the kitchen. Seasonal variation was important, with people feeling particularly “uneasy in hot seasons although they have to cook food” (FG2-P2). The problem of household air pollution in the hot season was felt to be exacerbated by having to use fans to keep cool.

A diverse range of other causes was perceived to be important, both inside and outside the home. In the house, women were aware that toilets were often not cleaned with water, and that this was problematic. Where toilets were absent, children sometimes urinated directly on the floor. Outside the house, women reported other “bad smells”—for example, food (vegetable peel, fruit, rice), animal dung, and urine—as sources of pollution. They discussed several circumstances in which such pollution was intensified. For example, it was reported that “bad smells” were exacerbated by adverse weather conditions, particularly in households with inadequate windows or doors. Other present but less important sources of pollution included dust and dirt being blown into the home, as well as mud and dust from non-brick houses. Dirt and bad smelling air were central to how air pollution was perceived. One woman said, “From the drain, when we close our window, then bad smells do not come, but when we open our window, then bad smell comes inside the house and make it more dirty” (FG1 P1).

### 3.1. Health Consequences of Air Pollution

**Figure 1:**
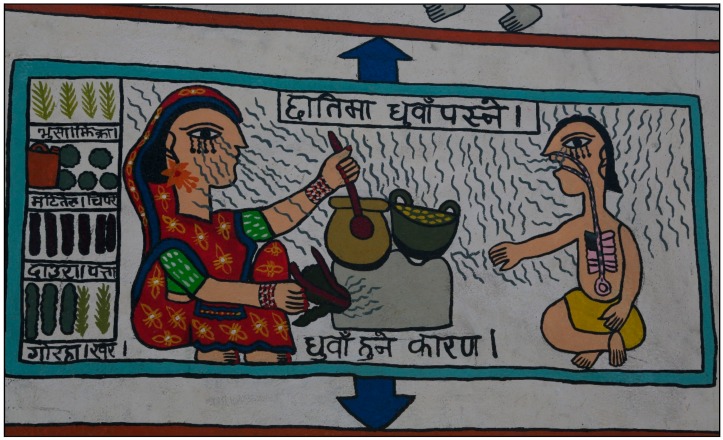
The effect of air pollution on the eyes and lungs. Translation: Top line = “smoke entering the chest. Bottom line = cause of smoke”. The picture shows some of the health effects of air pollution, including streaming of the eyes and damage to the lungs.

All of the participants acknowledged negative health effects of air pollution, although they emphasised them with varying strengths. Rather than focusing on respiratory health, the most commonly mentioned body part was the eye, with participants saying things like “eyes get swollen” (FG1 P1), “it causes pain in our eyes” (FG1 P3), or “the eyes become red” (FG1 P6). Figure 1 illustrates this effect of air pollution through the drawing of wiggly lines below the eyes of people in the drawing.

Air pollution was thought to lead to consequences such as itchiness, nausea, swelling, pain, irritation, redness, dizziness, and, in more extreme cases, transient visual impairment. The other main symptoms mentioned were breathing problems, fever, cough, weakness, runny nose, feeling faint, and head, neck, and chest pain, many of which are also visually represented in Figure 1. Some of the complaints were more vague, with one participant saying, “something troubling happens in my neck” (FG1 P2).

More generally, smoke was associated with a feeling of unease and restlessness. Participants reported a number of illnesses that they felt were associated with air pollution, such as whooping cough, asthma, tuberculosis, pneumonia, chest infections, stomach ulcers, cancer, diarrhoea, kidney disease, and heart disease. Pollution was also thought to increase the presence of flies and mosquitoes, leading indirectly to an increase in malaria and kala azar (visceral leishmaniasis).

**Figure 2:**
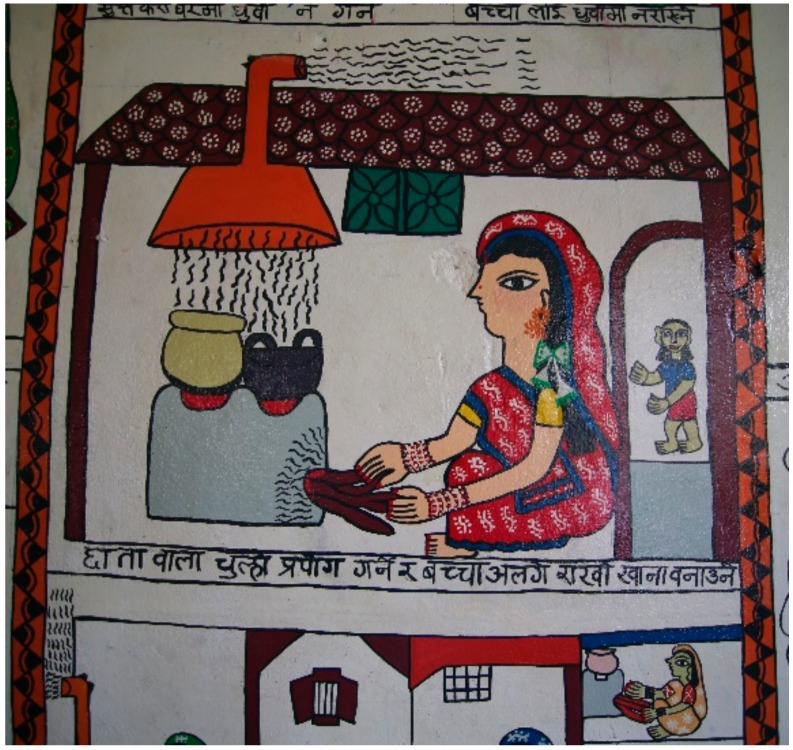
The perceived impact of air pollution on children and the need to keep them away from the cooking. Translation: Figure 2 = “Use of an umbrella-type exhaust above the cooking stove and keeping the child away”. This picture describes ways to reduce air pollution exposure. It shows an improved stove that has a chimney taking the smoke away and a child being kept in a different room.

**Figure 3:**
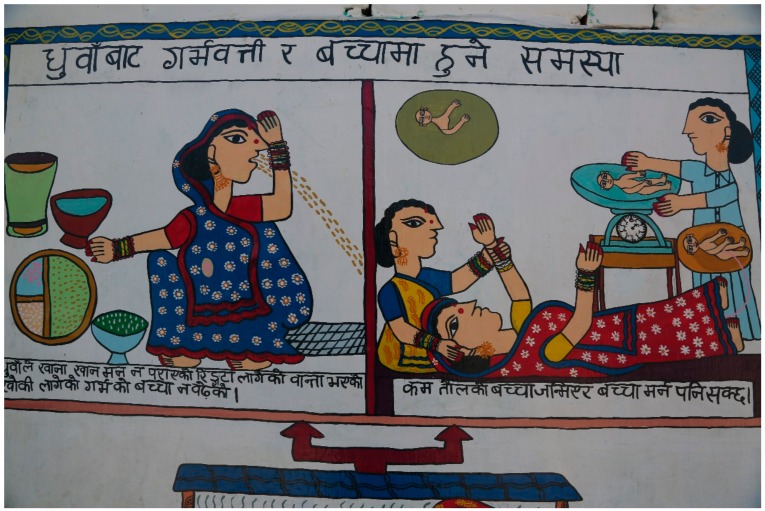
The effects of air pollution on women and newborn children. Translation: Top heading = Problems due to smoke in the mother and infant. Left photo = “Smoke causing poor appetite, giddiness, cough, and vomiting. Baby in utero not increasing in size”. The picture describes some of the health consequences for a pregnant woman, and the adverse effect on the growth of her fetus. Right photo = “Low birthweight baby may be born and may die as well”. This picture shows the result of air pollution exposure to the pregnant woman. It can lead to death of the fetus or newborn, or impair growth, leading to low birthweight.

Women were seen as particularly vulnerable to health-related harms, because they took primary responsibility for cooking; men spent much less time in the home. Children were thought to be even more vulnerable, particularly younger children who spent most of their time with their mothers. The smallest children could not leave the kitchen because of their dependence. Some mothers reported holding their children while cooking, resulting in exposure to more concentrated smoke. One participant said, “We are all big and can bear anything … but the children cannot bear it. The small children, from the time they are born, cannot bear that. Therefore, the children get sick immediately” (FG1 P7). Another said, “we have to save our children from the smoke” (FG2 P5). While cooking, another explicitly told her older children, “go now, and come back here when the food is cooked” (FG1 P3) to protect them as much as possible. Figure 2 illustrates the need to keep children away from cooking within the home. One of the artists in the group suggested a possible effect of pollution on the baby in the womb, although this was not mentioned by others. Figure 3 illustrates a newborn death and a baby being born with low birthweight.

**Figure 4:**
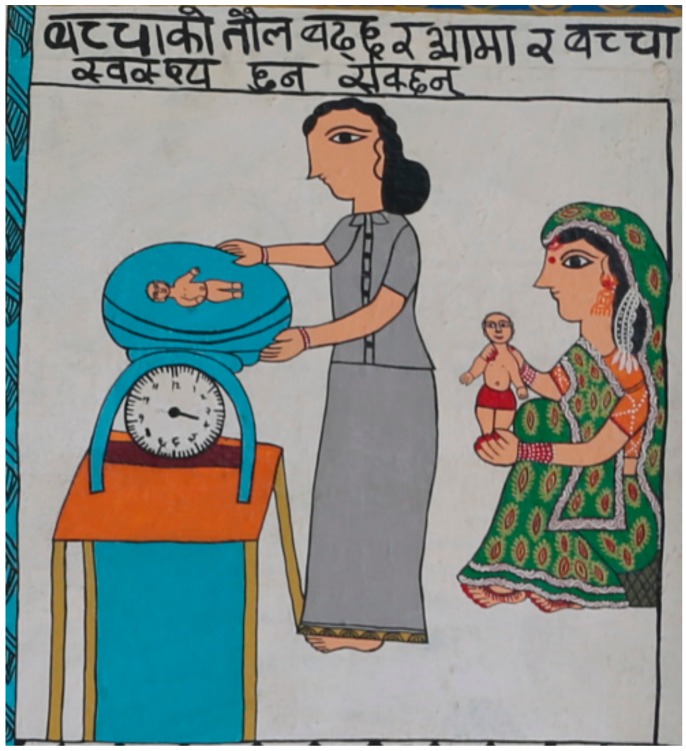
The health concerns in children. Translation: Figure 4 = “Baby’s weight increases and mother and baby may become healthy”. The picture shows a mother taking her child to be weighed by a health professional. It illustrates both her concern and also how weight can improve without exposure to air pollution.

Mothers also reported that their children complained about the effects of smoke. As with their mothers, eye problems were a major concern. Children were reported to generally get sick—cough and coryza were specifically mentioned—and to suffer from allergies, whooping cough, and pneumonia, which were all related to the smoke: “The symptoms shown in children like pneumonia, cough and fever” (FG4 P4); “Children having allergy from the dirtiness” (FG2 P5); and “By the blowing of straw and sand children get affected by having whooping cough and coryza” (FG3 P2). Figure 4 illustrates the health concerns about children’s exposure to air pollution by representing a visit to the doctor. Parents were strongly affected by illness in their children, and one participant said that the associated worry put her off eating.

### 3.2. Broader Health and Social Consequences of Air Pollution

Although most of the harms mentioned by participants were related to health, some additional negative consequences were noted. One woman said, “Polluted air inside the house can affect the children living inside it, and they aren’t able to perform at their best or pay attention to their education” (FG4 P1). It was felt that the associated sickness, particularly in children, prevented people from “eating the food nicely and … any kinds of work like in festivals such as Tihar … going to temple and doing worship or staying at home for watching TV” (FG1 P1).

### 3.3. Reduced Consumption of Food

Smoke could sometimes be so unbearable that a woman had to put out the fire and start cooking again, or, in the most severe cases, would simply not cook, going without food and only drinking water. This sentiment was echoed by several women, who said things such as, “for two out of three days I drink water only due to the smoke” (FG1 P1), “When we become worried, we do not eat food and take tension” (FG1 P4), and “Women go … without eating the food” (FG1 P3). The nauseating effects of air pollution and the effects on both women and their children are illustrated in Figure 3.

### 3.4. Loss of Finances and Time

Using biomass fuels was felt to be time-consuming in relation to cooking, cleaning surfaces covered in dust, and managing illnesses. One participant said, “The house looks black and dirty by cooking food as a result of the smoke” (FG3 P2). Dealing with smoke-related illness and lost productivity from cleaning were felt to be a significant financial drain. One participant said that, on dealing with air pollution related problems “by not spending money, we can save it for our children’s future” (FG4 P6).

Overall, there was acknowledgment that dealing with household air pollution had dramatic benefits and led to improvements in physical and mental health, productivity, and happiness.

### 3.5. Mitigation and Barriers to Change

#### Modification of Cooking Practice

**Figure 5:**
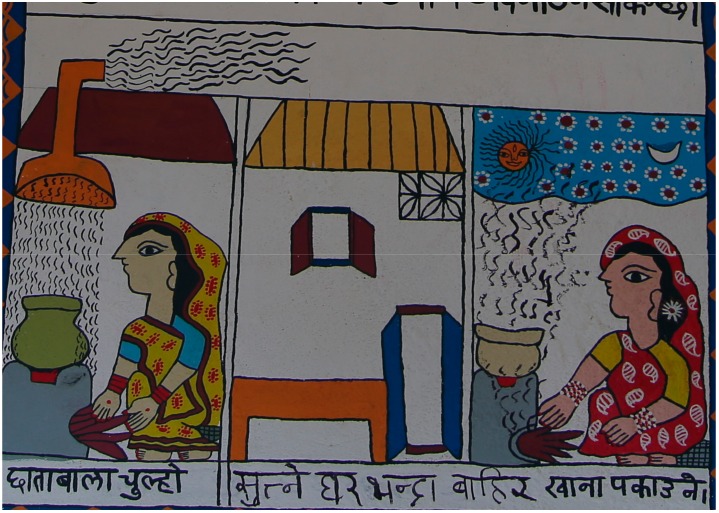
Potential solutions to reduce the air pollution. Translation: “Umbrella stove (hood). Cook outside the home (room) used for sleeping”. These pictures show methods to reduce air pollution exposure. On the left is a chimney or hood to channel the smoke outside. The middle picture shows increased ventilation by opening windows and doors. The right picture shows cooking outside.

Participants said that food should ideally be cooked quickly and the fire put out rapidly with water on completion, but the inherent limitations of biomass cookers meant that it was always a slow process. Using firewood instead of dung-cakes to start the fire was reported to lead to less smoke, and kerosene was felt to worsen the smoke. Puffing on the fire with a pipe (instead of by mouth) was seen as minimising smoke exposure for the person starting the fire. With regard to stove design, two feasible options suggested to reduce smoke were channeling it out via holes in the house (increasing ventilation) or via a chimney pipe, as illustrated in Figure 5.

One participant said that a doctor had recommended wearing spectacles to protect her eyes while cooking, and others mentioned wearing masks. In discussions about alternative stoves, heaters, coal stoves, and rice cookers were all mentioned. The most popular option was gas cookers, with many participants saying things like, “If cooking using gas, then there will be no smoke” (FG1 P1) or “we would like to cook the food using gas” (FG1 P3). However, there were concerns about accessing reliable gas, and about the cost of gas for poor communities, with one participant saying, “from where do poor people bring gas?” (FG1 P1).

### 3.6. Changes to Cooking Location and Improving Ventilation

There was a preference to have a kitchen outdoors, or on the roof (of concrete buildings), except during adverse weather conditions: “Cooking food outside. If there is a roof, then in roof. Now this is the room to cook food” (FG1 P3). Smoke was felt to be less concentrated in big open spaces (although there was a sense of being surrounded by smoke in large spaces), and “If there is no place to cook outside the house, then they have to cook food inside the house” (FG4 P2). The effects of smoke were thought to be greater in smaller, lower spaces. Other, more pragmatic, solutions involved only cooking on one side of the room and eating in a different room (or outside), away from where the smoke was most concentrated. However, it was acknowledged that smoke diffused throughout the house, and the practical benefit of cooking and eating in separate locations was limited. Some mothers stopped others from entering the kitchen whilst they cooked, although this was impractical for those looking after small children. Opening windows or doors was felt to help disperse smoke, particularly if kitchen windows could be opened, but this had the potential to make things worse if it was windy or if there was significant outdoor air pollution. Another strategy was to use fans to disperse the smoke. One woman discussed how she had made a structural change to her house by knocking down a wall to allow the smoke to escape.

### 3.7. Barriers to Dealing with Household Air Pollution

There was a consensus amongst the participants in one focus group that household air pollution was a problem, but that little had been done about it. The main reasons for this were insufficient money, or sometimes not knowing how to deal with the problem. This was especially so in small houses in which families lived, worked, slept, and cooked in the same room.

Feelings of helplessness were clear: “I am uneducated and I do not understand much. I just cook the food” (FG1 P2). Time to find a solution was an issue, particularly for mothers who had young children to look after. Many women commented on the need to prioritise other activities over those that would reduce air pollution. Comments were often fatalistic, such as saying that participants could not reduce the problem because “God has given us this air” (FG1 P5), and many said that they felt there was no effective solution. Certain challenges were thought to be very difficult to overcome; for example, ventilating the house, while knowing that opening windows lets in outdoor air pollution. The use of biomass stoves for heating during the winter may present a particular barrier to change. One participant said, “If we go near the fire in cold months … our house gets hot” (FG1 P1). However, this was only mentioned by one participant, with all of the other comments highlighting the negative aspects of this cooking method.

## 4. Discussion

The aim of our study was to gain a greater understanding of the meanings that women draw from their lived experience of air pollution associated with cooking in southern Nepal. The main strengths were the novel use of art combined with qualitative research techniques to understand participant perspectives. We think that this has enabled us to gain greater insight into the problems caused by air pollution and their potential solutions.

Generally, participants’ understanding of the health effects of air pollution was similar to the health effects described in the literature. Women identified air pollution as an important cause of ill health, and many said that reductions would lead to benefits in their lives. However, while they were aware of and concerned about a range of illnesses associated with air pollution, they prioritised them in specific ways. For example, the main concern was eye problems, rather than respiratory disease. As it stands, promoting household air pollution mitigation to reduce long-term outcomes such as chronic obstructive pulmonary disease may not be as motivating as promoting it to prevent eye problems. Importantly, in some cases, air pollution also led to the cessation of cooking. We were not able to quantify the extent of this problem, but it has potential nutritional implications for the family. We are not aware of this side effect of air pollution being reported previously.

Overall, participants saw biomass fuels as the main source of air pollution; however, contrary to our quantitative work, they perceived firewood as producing less smoke than dung, and that levels were higher in the summer, when combined with the hot weather and spread by fans. In this respect, they felt that heat exacerbated the air pollution, whereas our measurements showed much higher levels in the winter when heating was also prevalent [14]. Such dissonance between local perceptions and quantitative data needs to be addressed in order for interventions to be designed and implemented in acceptable and effective ways.

It was of interest that air pollution was conceptualised together with other “bad smells”. Opening windows would help reduce smoke, but it could also let in bad smells. Participants also felt that dirtiness and air pollution were synonymous, and that having a dirty house was also bad for health. This has implications for interventions that can tackle both. A combined approach that improves both hygiene and air quality could reduce multiple major infectious causes of child mortality such as pneumonia, diarrhoea, and vector-borne diseases.

### 4.1. Comparison with Similar Studies of Air Pollution

In general, similar types of qualitative studies have tended to focus on outdoor air pollution in high-income urban environments [31], where the causes and solutions are very different. Studies that have assessed perceptions of air pollution in LMICs have found mixed results regarding understanding of the health effects of air pollution. Muindi et al. conducted FGDs in Nairobi, Kenya, and Gordon et al. conducted FGDs in Ulan Baatar, Mongolia [32,33]. In both studies, people were generally aware of sources of household air pollution, but this did not necessarily change behaviours. Some barriers to change, such as cost and helplessness or fatalistic attitudes, were also similar to our study, but others were different. Contrary to our findings, in which the summer months were of more concern, air pollution was perceived to be much worse in winter in all cases—and especially in Ulan Baatar, where temperatures can fall to −40 °C. In line with our findings, the use of fires as a source of heat was important and considered beneficial. In Nairobi, eaves (ventilation ducts) were blocked, exacerbating household air pollution levels [32,33]. Person et al. conducted semi-structured interviews in rural Kenya, and noted similar health problems relating to the eyes and respiratory systems. A major problem was burns from the fire or ash. This was not reported by the women in our groups, though considering the types of stoves used, it is also likely to occur [34]. Edelstein et al. used questionnaires to interview women in Ethiopia. Similar health effects of air pollution were recognized, but the impact of air pollution on others, including children, was not mentioned in this Ethiopian study [35]. Rhodes et al. conducted in-depth interviews with adult women in Nepal, Peru, and Kenya prior to a stove intervention. Contrary to our findings, they found little knowledge of the health effects of smoke amongst their participants, but did note the context-specific nature of cooking practices, and the importance of engaging with the population in designing an intervention [36]. Sesan also noted the importance of dust and dirt collection in Kenya. In contrast to the health benefits, this was what led families to want separate kitchens. While it may improve exposure levels for other members of the family, it would not help those who do the cooking much [37].

### 4.2. Importance of Context-Relevant Interventions

A common intervention is improved stoves, but these may not be designed to meet local needs. They also may not yield sufficient financial or time savings, and there may be a mismatch between expectations and what is delivered [23]. Stanistreet et al. have summarised the factors associated with improved stove usage in LMICs. The reasons underlying successful stove interventions are complex, and encompass the individual, household, community, and national levels. Amongst those related to individuals and households, understanding the importance of culture and family/social life and cleanliness were considered important, and there was an appreciation of the health benefits of a new stove [21]. Involving community members in the research process makes it more likely that the right problems for them are identified and highlighted, which are issues that external researchers may not have considered important. The nuances in understanding and behaviour are context-specific, and solutions in one location may not work in another. While the cost of an improved stove is a potential barrier, it may be offset by the perceived benefits.

Two main ways to improve air quality would be to reduce the amount of smoke produced, with alternative fuels for cooking or improved stoves, or to reduce smoke exposure, ideally through chimneys or smoke hoods. Compared to the current scenario of cooking with biomass on open stoves, there was a clear preference for gas in all of the FGDs, and the main barrier to this appeared to be availability and cost. However, solutions to the household air pollution problem cannot be dissected from the context in which the problem occurs. This includes the need for heating and the broader concept of “bad smells”, which encompasses concerns about outdoor air pollution, as well as the general cleanliness of the home. These would need to be addressed, even if alternative cooking fuels could be provided. Any intervention is thus likely to be much more successful if it draws on a participatory approach or direct community involvement.

### 4.3. Limitations

Initially, we had hoped to involve men in the FGDs, in separate groups. This would have provided an important perspective, as men are often the head of the household, and the decision maker is often male in Southern Nepal. Unfortunately, it was not possible due to a lack of male volunteers. Future work addressing the understanding of men would be valuable. As we have highlighted, our study may have limited generalisability. While the problems faced in our population may be similar elsewhere, beliefs and barriers to change are likely to be context-specific. Similar qualitative work would be valuable to provide greater local understanding.

## 5. Conclusions

A number of research projects and programmes have been conducted to reduce the levels of household air pollution that women and other family members are exposed to, but few have asked those directly affected what the problems and solutions might be. Using a novel approach that combined FGDs with artwork, our study has summarised how women in southern Nepal interpret problems related to air pollution based on their lived experience. The in-depth insights gained can be important in the first step in the future design of locally appropriate and effective solutions.
